# Supplementary material for: Baseline Characterization of the Gut Microbiota of Field and Colony Populations of Phlebotomus tobbi and Preliminary Assessment of the Anti-Leishmanial Activity of Cultivable Bacteria
Source: Pathogens. 2026 Jun 23;15(7):658. doi: 10.3390/pathogens15070658 (PMC13416292; doi:10.3390/pathogens15070658)
Supplement: Supplementary file 1 [file pathogens-15-00658-s001.zip › pathogens-4357168-File S1/Sample_Information/absolute.abundance.html]

| sample\_id | customer\_label | Ct | gene\_copies\_per\_ul | genome\_copies\_per\_ul\* | DNA\_ng\_per\_ul\* |
| --- | --- | --- | --- | --- | --- |
| zr4724\_1V3V4 | Patient1 | 16.30 | 1742828 | 435707 | 2.2157242 |
| zr4724\_2V3V4 | Patient2 | 16.44 | 1599630 | 399908 | 2.0336736 |
| zr4724\_3V3V4 | Patient3 | 14.01 | 7084616 | 1771154 | 9.0069446 |
| zr4724\_4V3V4 | Patient4 | 15.88 | 2254033 | 563508 | 2.8656375 |
| zr4724\_5V3V4 | Patient5 | 14.86 | 4209644 | 1052411 | 5.3518822 |
| zr4724\_6V3V4 | Patient6 | 19.16 | 302407 | 75602 | 0.3844629 |
| zr4724\_7V3V4 | Patient7 | 20.39 | 142382 | 35596 | 0.1810182 |
| zr4724\_8V3V4 | Patient8 | 19.37 | 265913 | 66478 | 0.3380641 |
| zr4724\_9V3V4 | Patient9 | 16.05 | 2031169 | 507792 | 2.5823019 |
| zr4724\_10V3V4 | Patient10 | 20.00 | 180793 | 45198 | 0.2298478 |

  
\*Genome copies per microliter and DNA ng per microliter are calculated from the gene copies per microliter measured in the sample.
See the Materials & Methods section for more information.
